# Effects of IFN-γ coding plasmid supplementation in the immune response and protection elicited by *Trypanosoma cruzi* attenuated parasites

**DOI:** 10.1186/s12879-017-2834-6

**Published:** 2017-11-25

**Authors:** Cecilia Pérez Brandán, Andrea C. Mesías, Cecilia Parodi, Rubén O. Cimino, Carolina Pérez Brandán, Patricio Diosque, Miguel Ángel Basombrío

**Affiliations:** 1 Instituto de Patología Experimental, Consejo Nacional de Investigaciones Científicas y Técnicas (CONICET), Universidad Nacional de Salta, Salta-Capital, Argentina; 2 Instituto de Investigación de Enfermedades Tropicales, Sede Regional Orán. Universidad Nacional de Salta, Salta-Capital, Argentina; 3 Estación Experimental Agropecuaria Salta, Instituto Nacional de Tecnología Agropecuaria (INTA), Salta-Capital, Argentina

**Keywords:** *Trypanosoma cruzi*, IFN-γ, Attenuated infection

## Abstract

**Background:**

Previous studies showed that a naturally attenuated strain from *Trypanosoma cruzi* triggers an immune response mainly related to a Th2-type profile. Albeit this, a strong protection against virulent challenge was obtained after priming mice with this attenuated strain. However, this protection is not enough to completely clear parasites from the host. In *T. cruzi* infection, early Interferon-gamma (IFN-γ) is critical to lead type 1 responses able to control intracellular parasites. Therefore we evaluated whether the co-administration of a plasmid encoding murine IFN-γ could modify the immune response induced by infection with attenuated parasites and improve protection against further infections.

**Methods:**

C57BL/6J mice were infected intraperitoneally with three doses of live attenuated parasites in combination with plasmid pVXVR-mIFN-γ. Before each infection dose, sera samples were collected for parasite specific antibodies determination and cytokine quantification. To evaluate the recall response to *T. cruzi*, mice were challenged with virulent parasites 30 days after the last dose and parasite load in peripheral blood and heart was evaluated.

**Results:**

As determined by ELISA, significantly increase in *T. cruzi* specific antibodies response was detected in the group in which pVXVR-mIFN-γ was incorporated, with a higher predominance of IgG2a subtype in comparison to the group of mice only inoculated with attenuated parasites. At our limit of detection, serum levels of IFN-γ were not detected, however a slight decrease in IL-10 concentrations was observed in groups in which pVXVR-mIFN-γ was supplemented. To analyze if the administration of pVXVR-mIFN-γ has any beneficial effect in protection against subsequent infections, all experimental groups were submitted to a lethal challenge with virulent bloodstream trypomastigotes. Similar levels of challenge parasites were detected in peripheral blood and heart of mice primed with attenuated parasites alone or combined with plasmid DNA. Expansion of IgG antibodies was not significant in TCC+ pVXVR-mIFN-γ; however, the overall tendency to sustain a Th2 profile was maintained.

**Conclusions:**

Overall, these results suggest that administration of plasmid pVXVR-mIFN-γ could have beneficial effects on host specific antibody production in response to *T. cruzi* attenuated infection; however, this outcome is not reflected in an improved protection against further virulent infections.

**Electronic supplementary material:**

The online version of this article (10.1186/s12879-017-2834-6) contains supplementary material, which is available to authorized users.

## Background

*Trypanosoma cruzi* is a kinetoplastid parasite capable of infecting mammalian hosts leading in humans to the development of a number of clinical manifestations known as Chagas disease. This endemic disorder is of great importance in Central and South America since several million people are infected [1]. However, in recent years the increasing percentage of infected people in non-endemic areas due to migration influx has been a major focus of attention [2]. Chagas disease presents broad immunopathological profiles, ranging from asympthomatic cases; single digestive forms with megaesophagus and megacolon; single cardiac forms with intense myocarditis; or digestive and cardiac forms appearing together [3, 4]. Several years after infection, 30–40% of the infected persons develop progressive irreversible tissue damage. Currently, chemotherapy for Chagas disease patients is limited to the administration of benznidazole or nifurtimox. These drugs are highly effective in the acute phase, in congenital cases and in children with chronic infection, however; recent studies indicate a limitation in the efficacy of these drugs in chronic adults [5]. In most of the cases, people receiving drug therapy interrupt the treatment as a result of the severity of the side effects associated to nitro compounds [6]. Additionally, restriction of people living in endemic areas to the closer health centers deprives them from receiving a prompt diagnosis and the adequate treatment [7]. For many parasitic diseases, vaccines had the potential to overcome these misfortunes. Several immunoprophylactic as well as immunotherapeutic attempts have been made in order to prevent *T. cruzi* establishment and persistence in the host [8–12]. Still, to date, there is no vaccine licensed for Chagas disease, neither for humans nor for veterinary use.

In *T. cruzi* infection, the innate and adaptive immune responses play a crucial role in parasite control. These responses involve macrophages, natural killer (NK) cells, T and B lymphocytes, and the production of pro-inflammatory Th1 cytokines such as Interferon gamma (IFN-γ), Tumor Necrosis Factor Alpha (TNF-α) and interleukin 12 (IL-12) [13]. Also, the recognition of pathogen-associated molecular patterns (PAMP) by Toll Like receptors (TLRs), guides for activation of B and T cells, highlighting the important role of TLRs in connecting innate and acquired immunity [14]. IFN-γ is a key cytokine, secreted by NK cells and other cell types upon IL-12 generation, which has been shown to be crucial in guiding the development of naïve CD4^+^ T cells towards a Th1 phenotype as well as activating macrophages for the production of nitric oxide which is responsible for parasite clearance [15–17]. CD8^+^ T cells also secrete IFN-γ and their importance on parasite control has been well documented [18–20]. Still, it was recently shown that Th17 cells, another subset of CD4^+^ T cells, confer significantly stronger protection against *T. cruzi* than even Th1 cells [21]. In any event, the role of IFN-γ in controlling intracellular parasite infections is crucial. Despite the response orchestrated, *T. cruzi* can induce IL-10 production by dendritic cells and persist indefinitely in the host [22, 23].

It has been described that the naturally attenuated TCC strain from *T. cruzi* triggers a Th2-type immune profile in contraposition to virulent strains which are mainly related to a Th1-type one [24]. Albeit the Th2-type outline triggered by TCC infection, this attenuated strain has been extensively used by our group in prime/boost/challenge experiments [25–31]. This evaluation was also extended to genetically modified mutant clones from this same strain [32, 33]. Even though protection against further infections is strong enough to control parasite replication, is not sufficient to completely clear the parasites from the host. With this in mind we wondered if the addition of an external source of IFN-γ could change or improve the immunological and protective effect of attenuated TCC parasites. In this work, we describe the enhancement in the specific antibody responses of mice after infection with TCC attenuated parasites co-administered with plasmid DNA encoding murine IFN-γ. However, this upgrade in the particular humoral response elicited did not correlate with a significant reduction in parasite burden after challenge with virulent parasites.

## Methods

### Parasites and plasmid preparation

For the infection assays, metacyclic trypomastigotes from the naturally attenuated TCC strain from *T. cruzi* were used [34]. Briefly, epimastigotes were grown at 28 °C in liver digested neutralized tryptose medium (LDNT), supplemented with 10% fetal bovine serum (FBS) until they reached log-phase. Metacyclics trypomastigotes were then obtained by epimastigotes differentiation under chemically defined conditions (TAU 3AAG medium). Complement resistant forms were purified using normal non decomplemented serum, quantified in a Neubauer chamber and further used to inoculate experimental animals. Virulent blood trypomastigotes forms from the Tulahuen strain were prepared by routinely biweekly passage through BALB/c mice. The eukaryotic expression plasmid pVXVR-mIFN-γ was gently donated by Dr. Gomez Hernandez from Brazil. Concisely, mIFN-γ was amplified from splenocytes cDNA previously stimulated with lipopolysaccharides (LPS) and then cloned in pVXVR plasmid, a fusion between plasmids pCDNA3 and VR1012. Final construction, pVXVR-mIFN-γ was transformed into *Escherichia coli* DH5α competent cells, grown in LB media containing 100 μg/ml ampicillin, and purified by anion-exchange chromatography using the Endo-free Qiagen maxiprep kit (Qiagen) as per manufacturer recommendations and then quantified using Nanodrop 2000 (Thermo Scientific).

### Infection in mice

Six to 8 week old male C57BL/6 J mice were used throughout these studies. Parasites and plasmid DNA were administered intraperitoneally (IP). For this, 10^5^ purified metacyclic trypomastigotes/mouse from the TCC strain were used and/or 50 μg/mouse of plasmid pVXVR-mIFN-γ according to the particular experiment. Three doses were delivered at 4-week intervals. Control groups consisting of empty plasmid and non-infected animals were included in the experiment.

### *T. cruzi*-specific IgGs ELISA

During the infection period with attenuated parasites and plasmid DNA, serum samples were collected and aliquots were maintained at −80 °C until use for IgGs determination. Total immunoglobulin G antibodies (1:2500 dilution-SIGMA-ALDRICH) against *T. cruzi* were measured by the enzyme-linked immunosorbent assay (ELISA) using *T. cruzi* epimastigote homogenate as antigen (1 μg/100 μl-Coating Buffer/well). To identify the antibody subtypes, plates were coated as above, blocked with Phosphate Buffer Solution (PBS) 5% non-fat dry milk, and then incubated with serum samples (1∶100 dilution, 100-μl/well) for 1 h, biotin-conjugated goat anti-mouse Ig subtypes (IgG1 or IgG2a-1:3500 dilution-BD Pharmigen) for 1 h, and streptavidin-horseradish peroxidase conjugate (BD-Pharmigen) for 1 h at 37 °C. Color was developed with TMB Substrate Reagent Set (BD-Pharmigen) and monitored at 450 nm using a Tecan Infinite Pro microplate reader.

### Spleen cells culture supernatant

Spleens were removed from euthanized infected mice, macerated on a sterile mesh, and cells were resuspended in RPMI 1640 medium. Following centrifugation at 160×g for 10 min at 4 °C, the cells were resuspended in a lysis solution (0.17 M Tris pH 7.2, 0.16 M NH_4_Cl) to remove erythrocytes. The remaining splenocytes were washed three times with RPMI and resuspended in RPMI supplemented with 20 mM glutamine, 10% NaHCO_3_ and 10% fetal bovine serum. Viability of cells was assessed by Trypan blue exclusion and cell number was determined in a Neubauer chamber. Splenocytes (10^6^cells/well in triplicate) were cultured for 48 h at 37 °C and 5% CO_2_, with or without stimulation with 25 μg/mL of *T. cruzi* epimastigote homogenate or with 50 μg/mL of Phytohaemagglutinin (PHA). Cell culture medium was then collected and aliquots stored at −80 °C until use for cytokines determination.

### Cytokine response

Sera samples as well as splenocytes culture supernatant were used for cytokines’ measurement (IL-10, TNF-α and IFN-γ) using optEIA enzyme-linked immunosorbent assay (ELISA) kits (BD-Pharmingen), according to the manufacturer’s specifications.

### Survival rate and parasitemia

To evaluate the recall response to *T. cruzi*, mice were challenged with *T. cruzi* Tulahuen strain (500 blood trypomastigotes/mouse, IP). Biweekly, blood (10 μl) was drawn from the tail tip of mice under slight anesthesia, and the number of parasites per 100 fields (parasitemia) was recorded from fresh blood mounts under microscope (×400). Mortality was checked daily until 30 days post challenge and expressed as percentage of survival.

### Tissue parasite burden

Thirty days post virulent challenge mice were sacrificed by carbon dioxide (CO_2_) overexposure. At this point serum samples were taken for determining IgGs expansion and cytokines responses. Heart samples were taken for parasite DNA quantification. Total DNA from heart tissue (50 mg) was isolated using the ADN-Puriprep Highway nucleic acid kit (InbioHighway), according to instructions provided by the manufacturer. Total DNA (10 ng) was used as a template, and real-time PCR performed on a LineGene 9640 Sequence Detection System (BIOER) with SYBR Green Supermix (Biosystems) and Tc18SrDNA-specific oligonucleotides (SAT_F 5’GCAGTCGGCKGATCGTTTTCG-3′ and SAT_R 5’TTCAGRGTTGTTTGGTGTCCAGTG-3′). Data were normalized to murine TNF-α (TNF_F 5′-TCCCTCTCATCAGTTCTATGGCCCA-3′ and TNF_R 5’CAGCAAGCATCTATGCACTTAGACCCC-3′).

### Statistical analysis

Continuous variables, such as antibody levels and parasite concentrations in blood samples, were analyzed with the two-tailed Wilcoxon signed-rank test for time course plots and with the Mann-Whitney or Kruskal-Wallis test for single-day measurements with Dunn’s Multiple Comparison Test. Values are expressed as mean with standard errors of the mean (SEM) from at least three independent experiments. Differences between two groups were considered significant and are shown by *p˂0.05, **p˂0.01, ***p˂0.001.

## Results

### IFN-γ production alters the specific immune response elicited by attenuated parasites

**Fig. 1:**
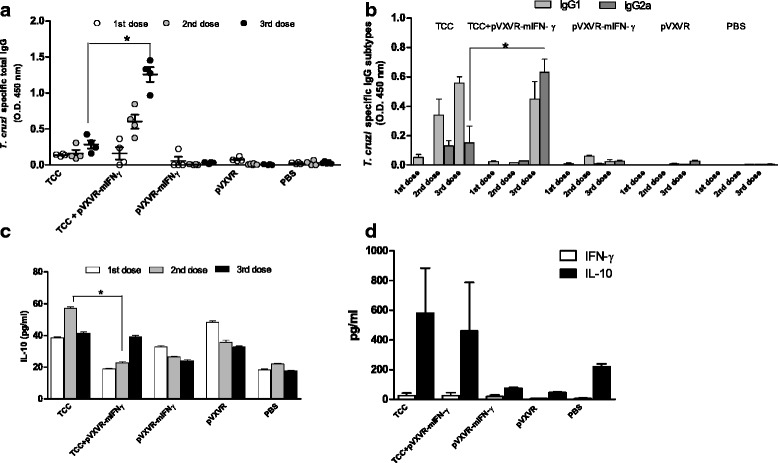
pVXVR-mIFN-γ administration alters the parasite-specific immune response elicited by infection with attenuated parasites. Mice (*n* = 4) were infected (4 weeks apart) with 3 doses of 10^5^ metacyclic trypomatigotes of the attenuated TCC strain, 50 μg of plasmid pVXVR-mIFN-γ or a combination of both. After each infection dose, serum samples were collected for IgG and cytokines determination. **a** *T. cruzi* specific total IgGs levels; **b** serum levels of parasite specific IgG subtypes, **c** IL-10 measurement determined by ELISA and (**d**) IL-10 and IFN-γ levels in supernatant from *T. cruzi* stimulated spleen cells. Data are representative of three independent experiments. * p < 0.05 Kruskal-Wallis test for single-day measurements with Dunn’s Multiple Comparison Test

To determine the levels of *T. cruzi*-specific IgGs after infection with attenuated parasites, serum samples were collected 30 days after each dose. As determined by ELISA, parasite specific antibodies levels increased after each boost and were significantly elevated in the group in which pVXVR-mIFN-γ was co-administered in combination with attenuated parasites when compared to the TCC infected group (Fig. 1a, *p < 0.05). To indirectly evaluate the impact of pVXVR-mIFN-γ administration on the effector cytokine pattern during infection with attenuated parasites, the levels of parasite specific IgG subtypes were determined by ELISA. IgG1 antibodies were primarily detected in TCC-infected animals with significantly low predominance of IgG2a (Fig. 1b); however when pVXVR-mIFN-γ plasmid was administered, a balance between IgG1 and IgG2a was observed with a significantly increase in anti-*T. cruzi* IgG2a antibodies when compared to TCC-infected animals (Fig. 1b, *p < 0.05, TCC vs TCC+ pVXVR-mIFN-γ). Because the switch towards a different subtype could be promoted by various cytokines, serum concentrations of regulatory (IL-10) and pro-inflammatory (IFN-γ) cytokines were measured. We observed that sustained boosts with TCC parasites combined with plasmid DNA resulted in increased production of IL-10. However, when pVXVR-mIFN-γ is added, the concentration of IL-10 at different time points tended to be slightly lower (Fig. 1c, *p < 0.05). IFN-γ levels were found below the detection limit of the technique in all of the tested samples. Spleen cells from TCC and TCC + pVXVR-mIFN-γ infected animals were also analyzed. Cells were stimulated for 48hs with *T. cruzi* antigen and the levels of IFN-γ and IL-10 were determined in culture supernatant (Fig. 1d). As in serum samples, IFN-γ was not detected at significant levels. IL-10 production by splenocytes was similar in TCC and in TCC + pVXVR-mIFN-γ infected animals but different from pVXVR-mIFN-γ administered animals or naïve ones. Interesting to note is that naïve animals exhibited a lower level of IL-10 compared to animals receiving pVXVR-mIFN-γ possibly indicating that the presence of the IFN-γ plasmid could be rendering for a lower production of IL-10 by spleen cells. From these results is evident to conclude that the addition of pVXVR-mIFN-γ may favor the expansion of the specific IgG response mounted by the prime infection with TCC attenuated parasites. Even more, a tendency in altering the IgG subtype levels (increased in IgG2a profile) and IL-10 production was observed after plasmid addition, indicating that it would be possible to redirect the Th2-type phenotype obtained by the infection with TCC attenuated parasites towards a mixed Th1/Th2 profile.

### TCC + pVXVR-mIFN-γ elicited protection against in vivo lethal challenge and parasite replication in heart

**Fig. 2:**
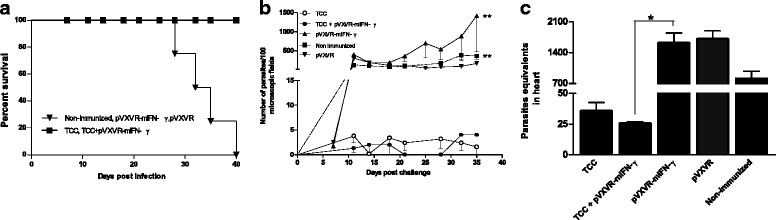
Control of parasite replication in animals primed with TCC+ pVXVR-mIFN-γ after lethal challenge. Thirty days after the last dose with attenuated parasites and plasmid DNA, animals were challenged with 500 blood trypomastigotes from the Tulahuen strain (n = 4). **a** Survival rate during the challenge period; **b** parasite load in peripheral blood as determined by fresh blood mount and (**c**) parasite load in heart as determined by Real Time PCR 30 days post challenge. Data are representative of three independent experiments. * p < 0.05 and ** p < 0.01 Kruskal-Wallis test for single-day measurements with Dunn’s Multiple Comparison Test

To analyze if the immune response elicited by the administration of plasmid pVXVR-mIFN-γ in conjunction with live attenuated parasites was protective, all experimental groups were submitted to a lethal challenge with virulent bloodstream trypomastigotes. Animals were infected 30 days after the last dose with attenuated parasites alone or in combination with plasmid pVXVR-mIFN-γ and parasites level recorded every week during the acute phase. Survival was monitored daily and all mice from control groups succumbed to death after day 30 post challenge (Fig. 2a). Meanwhile, TCC and TCC + pVXVR-mIFN-γ groups showed 100% survival until the end of follow-up. The level of circulating parasites in peripheral blood from TCC or TCC + pVXVR-mIFN-γ infected animals was scarcely detectable (Fig. 2b). In this model and with this technique, the advantage of administrating pVXVR-mIFN-γ or not was not evident since no statistically significant differences were found between both groups. However, parasitemia from these groups was significantly different from the control groups (Fig. 2b, **p < 0.01). Real time PCR outcome verified the above mentioned results, since a significant reduction in parasite-specific Tc18SrDNA signal in heart of TCC and TCC + pVXVR-mIFN-γ infected animals was detected when compared to control groups (Fig. 2c, *p < 0.05 in TCC and TCC+ pVXVR-mIFN-γ vs pVXVR-mIFN-γ). Also with this technique the advantages of introducing pVXVR-mIFN-γ were not evident since no significant differences with TCC primed animals were detected.

### Parasite specific immune response is expanded in reply to *T. cruzi* virulent infection

**Fig. 3:**
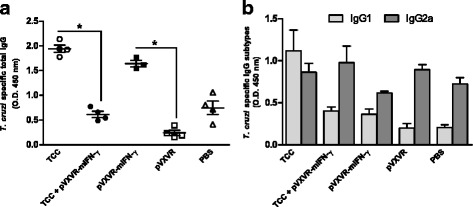
Expansion of immune specific antibodies in response to *T. cruzi* infection. Thirty days after challenge serum samples from animals primed with TCC, TCC+ pVXVR-IFN-γ, pVXVR-IFN-γ and PBS were collected for (**a**) parasite specific IgG antibodies and (**b**) IgG subtypes by ELISA. Data are representative of three independent experiments. * p < 0.05 Kruskal-Wallis test for single-day measurements with Dunn’s Multiple Comparison Test

**Fig. 4:**
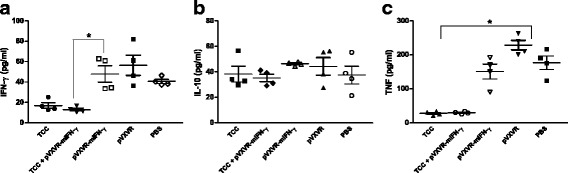
Cytokines levels in response to virulent *T. cruzi* infection. Cytokine concentration in serum of TCC and TCC+ pVXVR-IFN-γ primed animals challenged with virulent parasites. Samples were collected 30 days post challenge and cytokines level measured by ELISA for (**a**) IFN-γ, (**b**) IL-10 and (**c**) TNF-α. Data are representative of three independent experiments. * p < 0.05 Kruskal-Wallis test for single-day measurements with Dunn’s Multiple Comparison Test

In order to evaluate the antibody response elicited in primed animals after challenge with virulent parasites, serum samples were collected 30 days post infection. TCC infected animals exhibited a significant expansion of parasite specific IgG in response to *T. cruzi* virulent infection (Fig. 3a, *p < 0.05). However, in the case in which pVXVR-mIFN-γ was co-administered together with attenuated parasites, expansion was significantly diminished. Although not significant, the IgG1 response in TCC + pVXVR-mIFN-γ primed/challenged animals was lower than in TCC primed/challenged group. IgG2a was to some extent expanded in the case in which pVXVR-mIFN-γ was added, and diminished in TCC-primed mice (Fig. 3b). These data suggested that TCC-primed elicited a parasite-specific IgG response upon challenge infection and that addition of plasmid pVXVR-mIFN-γ, even not contributing to this expansion, may favor the guidance towards a Th1 phenotype. Serum concentrations of IL-10, TNF-α and IFN-γ cytokines were also measured after virulent challenge. The levels of IFN-γ and TNF-α were similar in TCC or TCC + pVXVR-IFN-γ primed/challenged animals in comparison to non-primed animals (Fig. 4a-c). However, unchanged values for IL-10 production were detected among all experimental groups (Fig. 4b).

## Discussion

It is known that during the course of many infections, initial IFN-γ production is fundamental and may be important in the development of resistance to many intracellular infections such as the one produced by *T. cruzi*. Also, the activation of the specific cells responsible for the production of this cytokine requires the presence of live parasites [35]. In an attempt to elucidate if parasite specific immune response could be modified or improved, we decided to co-administer, together with highly attenuated parasites, a eukaryotic expression plasmid encoding the murine sequence of the cytokine IFN-γ. Our principal observation is that the addition of the plasmid containing IFN-γ significantly improves the generation of parasite specific antibodies in response to prime infection. It is well known that early secreted IFN-γ by NK cells activates plasma B cells to produce antibodies in order to lyse extracellular trypomastigotes or to facilitate the opsonization process. Therefore the effect of early introducing a source of IFN-γ fostering the natural generation of parasite specific antibodies upon infection is beneficial as demonstrated by our results. The importance of IFN-γ in the generation of a Th1 response and in the formation of IFN-γ-dependent IgG2a antibodies has been demonstrated since parasite specific antibody response of infected IFN-γ-KO mice was restricted to IgG1 antibodies, being parasite specific IgG2a antibodies absent [36]. TCC-primed animals presented a larger predominance of IgG1 rather than IgG2a antibodies; however, with IFN-γ plasmid administration, this phenotype could be slightly biased towards a Th1/Th2 balance with reduced values of IgG1a and enlarged levels of IgG2a. This switch of Ig-isotype to cytophilic and complement-fixing IgG2a antibodies has also been demonstrated to be induced by IFN-γ [37]. Even more, these same results were observed in other infections assays following the same experimental design, in which attenuated parasites and plasmid DNA were administered oral or intramuscularly, indicating that the administration route, especially for plasmid DNA, does not modify the results herein presented (Additional file 1: Figure S1).

When a host has been immunized, either by infection or vaccination, one of the most important parameters to be measured is its capacity to respond against a subsequent infection. All immunized animals were protected against death, with 100% of these mice surviving the lethal challenge. Virulent infection of primed animals with a lethal dose of bloodstream trypomastigotes led to significantly decreased parasitemias and parasite load in heart samples in all the groups infected with attenuated parasites, regardless administration or not of plasmid coding for IFN-γ. Interesting to note is that previous additions of pVXVR-mIFN-γ solely and later infection with virulent parasites resulted, in some extent, in exacerbated parasite load in blood as well as in heart samples, suggesting that unregulated IFN-γ inner production could be unfavorable for the host. These results are supported by the observation that parasite load in mice inoculated with naked plasmid and later infected with virulent parasites resulted in values similar or lower than the control group inoculated with PBS and challenged later (data not shown). All together, these results indicate that infection with attenuated parasites per se is efficient in regulating parasite burden in target organs and peripheral blood and, that the combined administration of pVXVR-mIFN-γ has no significant role in helping controlling parasite replication at this time point.

Challenged infection resulted in a rapid and potent expansion of parasite specific antibody response in TCC-infected animals; however this expansion is not favored by pVXVR-mIFN-γ addition. One possible explanation for this observation maybe that upregulation of FcR by IFN-γ activated macrophages [38] is augmented by an additional source of IFN-γ, increasing the captured of opsonized parasites, therefore requiring the availability of a larger amount of parasite specific antibodies. Nevertheless, a considerable level of *T. cruzi* specific antibodies was detected in the group of animals primed with pVXVR-mIFN-γ plasmid alone, indicating that mice which have never seen *T. cruzi* antigen before but were stimulated with plasmid carrying IFN-γ posses the capacity to better respond, at least at the humoral level, to a subsequent virulent infection. This last result is also supported by the fact that animals primed with empty plasmid and later challenged with virulent parasites presented antibodies levels similar to control group inoculated with PBS. The predominance of a Th1 phenotype in expanded specific IgGs is maintained by pVXVR-mIFN-γ supply during infection with attenuated parasites, as well as after challenge infection. Since it was reported that IgG2a antibodies efficiently mediates clearance of blood trypomastigotes [39], the increase in anti–*T. cruzi* IgG2a subtype antibodies after challenge of primed animal could be important to reduce parasite proliferation. Additionally, the assessment of the cytokines profile also showed differences among groups. At 30 days post challenge, the levels of IFN-γ and TNF-α in primed animals were lower in comparison to non-primed animals (pVXVR and PBS group). This finding was in concordance with the level of circulating parasites in peripheral blood at this time point, suggesting that IFN-γ and TNF-α production in TCC and TCC + pVXVR- IFN-γ primed-animals was not longer necessary since parasite clearance already occurred. The elevated levels of TNF-α found within the non-primed groups could have a harmful effect in the host, exacerbating the existing inflammatory response. It has been demonstrated that TNF-α increased during early *T. cruzi* infection is associated to the induction of trypanomicide mechanisms and tissue injury [40]. In this regard, we suggest that a modulated amount of IFN-γ and TNF-α secreted after a virulent challenge could be related to a controlled response against the antigen, meanwhile, an exacerbation of this response with elevated levels of both cytokines, as we have shown in the control groups could be responsible for inflammation and consequent tissue damage. Interestingly, in our experiments, IL-10 production did not differ among groups. Similar results were observed by others, in which chronically infected/challenged mice only showed IL-10 production after day 7 post challenge, reaching later, to levels similar to those found in non infected/challenged mice. These authors suggest that IL-10 production, as IFN-γ production of chronically infected mice depends on intense proinflammatory signals induced by parasites [41].

Besides stimulating B cells, perhaps the most noticeable effect that IFN-γ exerts is the enhancement of microbicidal activity by stimulating macrophages to produce Nitric Oxide (NO) and kill intracellular amastigotes. This evidence has recently been confirmed by means of “Macrophage Insensitive to Interferon Gamma” KO mice, since macrophages from these animals were unable to produce NO and kill intracellular parasites upon challenge and after priming with IFN-γ [42]. On the other hand, the impact of IFN-γ in the modulation of chemokine expression in vivo has awakened attention [43]. Therefore, it is quite obvious to postulate that other immune mechanisms, beyond the ones herein studied, might had taken place during this immunization process leading to an improved activation and priming of immune specific cells capable of controlling parasite spread.

## Conclusions

In summary we conclude that TCC + pVXVR-mIFN-γ infection successfully primed B cells and induced high parasite specific antibody response with a Th1/Th2 phenotype able to protect against a virulent challenge. However, this increase in the specific antibody response, due to pVXVR-mIFN-γ addition, is not enough to improve the control of parasite replication and persistence in the host. Basic immunological studies indicate that antibodies solely are not enough to manage *T. cruzi* infection and that parasite control relies mostly on a cellular immune response with predominance of activation of CD8^+^ T cells [18, 19, 44]. In a previous work we showed that CD8^+^ T cells were induced by infection with TCC attenuated parasites [32] thus we supposed that this response could be very likely strengthened by endogenous IFN-γ production and expanded after virulent challenge. Licensed vaccines against many pathogens confer protection by stimulating B cells to produce a wide repertoire of pathogen-specific antibodies. Our most striking observation herein presented, that *T. cruzi*–specific humoral responses elicited by attenuated parasites can be optimized and presumably switch towards a Th1/Th2 balance by plasmid encoding IFNγ supplementation, addresses the possibility that other immune-boosting strategies, for therapeutic or immuneprophylactic vaccines approaches, should be deeper inquired into. The results herein presented emphasized the fact that a *T. cruzi* vaccine should be addressed from a multicomponent point of view, properly activating the humoral and cellular responses needed for protection.

## Additional file

Additional file 1: Figure S1. pVXVR-mIFN-γ administration alters the parasite-specific immune response elicited by infection with attenuated parasites by the intramuscular and oral route. Mice (*n* = 4) were infected (4 weeks apart) with 3 doses of 10^5^ metacyclic trypomatigotes of the attenuated TCC strain, 50 μg of plasmid pVXVR-mIFN-γ or a combination of both by the intramuscular (A-C) or oral (B-D) route. After each infection dose, serum samples were collected for (A-B) *T. cruzi* specific total IgGs levels and (C-D) serum levels of parasite specific IgG subtypes. (PDF 48 kb)

## Abbreviations

CO_2_: Carbon dioxide
ELISA: Enzyme-linked immunosorbent assay
FBS: Fetal bovine serum
IFN-γ: Interferon gamma
IL: Interleukin
IP: Intraperitoneally
LDNT: Liver digested neutralized tryptose medium
LPS: Lipopolysaccharides
NK: Natural killer
NO: Nitric Oxide
PAMP: Pathogen-associated molecular patterns
PBS: Phosphate Buffer Solution
PHA: Phytohaemagglutinin
SEM: Standard errors of the mean
TLRs: Toll Like receptors
TNF-α: Tumor Necrosis Factor Alpha

## References

[1] Stanaway JD, Roth G (2015). The burden of Chagas disease: estimates and challenges. Glob Heart.

[2] Liu Q, Zhou XN (2015). Preventing the transmission of American trypanosomiasis and its spread into non-endemic countries. Infect Dis Poverty.

[3] Bilate AM, Cunha-Neto E (2008). Chagas disease cardiomyopathy: current concepts of an old disease. Rev Inst Med Trop Sao Paulo.

[4] Coura JR, Borges-Pereira J (2010). Chagas disease: 100 years after its discovery. A systemic review. Acta Trop.

[5] Pecoul B, Batista C, Stobbaerts E, Ribeiro I, Vilasanjuan R, Gascon J, Pinazo MJ, Moriana S, Gold S, Pereiro A (2016). The BENEFIT trial: where do we go from here?. PLoS Negl Trop Dis.

[6] Viotti R, Vigliano C, Lococo B, Alvarez MG, Petti M, Bertocchi G, Armenti A (2009). Side effects of benznidazole as treatment in chronic Chagas disease: fears and realities. Expert Rev Anti-Infect Ther.

[7] Pinheiro E, Brum-Soares L, Reis R, Cubides JC (2017). Chagas disease: review of needs, neglect, and obstacles to treatment access in Latin America. Rev Soc Bras Med Trop.

[8] Perez Brandan C, Basombrio MA (2012). Genetically attenuated *Trypanosoma cruzi* parasites as a potential vaccination tool. Bioengineered.

[9] Quijano-Hernandez I, Dumonteil E (2011). Advances and challenges towards a vaccine against Chagas disease. Hum Vaccin.

[10] Rodriguez-Morales O, Monteon-Padilla V, Carrillo-Sanchez SC, Rios-Castro M, Martinez-Cruz M, Carabarin-Lima A, Arce-Fonseca M (2015). Experimental vaccines against Chagas disease: a journey through history. J Immunol Res.

[11] Sanchez-Valdez FJ, Perez Brandan C, Ferreira A, Basombrio MA (2015). Gene-deleted live-attenuated *Trypanosoma cruzi* parasites as vaccines to protect against Chagas disease. Expert Rev Vaccines.

[12] Vazquez-Chagoyan JC, Gupta S, Garg NJ (2011). Vaccine development against Trypanosoma cruzi and Chagas disease. Adv Parasitol.

[13] Junqueira C, Caetano B, Bartholomeu DC, Melo MB, Ropert C, Rodrigues MM, Gazzinelli RT (2010). The endless race between *Trypanosoma cruzi* and host immunity: lessons for and beyond Chagas disease. Expert Rev Mol Med.

[14] Rodrigues MM, Oliveira AC, Bellio M (2012). The immune response to *Trypanosoma cruzi*: role of toll-like receptors and perspectives for vaccine development. J Parasitol Res.

[15] Antunez MI, Cardoni RL (2000). IL-12 and IFN-gamma production, and NK cell activity, in acute and chronic experimental *Trypanosoma cruzi* infections. Immunol Lett.

[16] Gutierrez FR, Mineo TW, Pavanelli WR, Guedes PM, Silva JS (2009). The effects of nitric oxide on the immune system during *Trypanosoma cruzi* infection. Mem Inst Oswaldo Cruz.

[17] Vespa GN, Cunha FQ, Silva JS (1994). Nitric oxide is involved in control of *Trypanosoma cruzi*-induced parasitemia and directly kills the parasite in vitro. Infect Immun.

[18] Padilla AM, Bustamante JM, Tarleton RL (2009). CD8^+^ T cells in *Trypanosoma cruzi* infection. Curr Opin Immunol.

[19] Tarleton RL (2015). CD8+ T cells in *Trypanosoma cruzi* infection. Semin Immunopathol.

[20] Vasconcelos JR, Dominguez MR, Neves RL, Ersching J, Araujo A, Santos LI, Virgilio FS, Machado AV, Bruna-Romero O, Gazzinelli RT (2014). Adenovirus vector-induced CD8(+) T effector memory cell differentiation and recirculation, but not proliferation, are important for protective immunity against experimental *Trypanosoma cruzi* infection. Hum Gene Ther.

[21] Cai CW, Blase JR, Zhang X, Eickhoff CS, Hoft DF (2016). Th17 cells are more protective than Th1 cells against the intracellular parasite *Trypanosoma cruzi*. PLoS Pathog.

[22] Fernandes MC, Andrews NW (2012). Host cell invasion by *Trypanosoma cruzi*: a unique strategy that promotes persistence. FEMS Microbiol Rev.

[23] Nagajyothi F, Machado FS, Burleigh BA, Jelicks LA, Scherer PE, Mukherjee S, Lisanti MP, Weiss LM, Garg NJ, Tanowitz HB (2012). Mechanisms of *Trypanosoma cruzi* persistence in Chagas disease. Cell Microbiol.

[24] Revelli S, Gomez L, Wietzerbin J, Bottasso O, Basombrio MA (1999). Levels of tumor necrosis factor alpha, gamma interferon, and interleukins 4,6, and 10 as determined in mice infected with virulent or attenuated strains of Trypanosoma cruzi. Parasitol Res.

[25] Basombrio MA (1990). *Trypanosoma cruzi*: partial prevention of the natural infection of guinea pigs with a killed parasite vaccine. Exp Parasitol.

[26] Basombrio MA, Arredes H (1987). Long-term immunological response induced by attenuated *Trypanosoma cruzi* in mice. J Parasitol.

[27] Basombrio MA, Arredes H, Uncos DA, Rossi R, Alvarez E (1987). Field trial of vaccination against American trypanosomiasis (Chagas' disease) in domestic guinea pigs. Am J Trop Med Hyg.

[28] Basombrio MA, Arredes HR, Rossi R (1986). Molina de Raspi E: Histopathological and parasitological evidence of immunization of mice against challenge with 17 wild isolates of Trypanosoma cruzi. Int J Parasitol.

[29] Basombrio MA, Besuschio S (1982). *Trypanosoma cruz*i culture used as vaccine to prevent chronic Chagas' disease in mice. Infect Immun.

[30] Basombrio MA, Besuschio S, Cossio PM (1982). Side effects of immunization with liver attenuated *Trypanosoma cruzi* in mice and rabbits. Infect Immun.

[31] Basombrio MA, Segura MA, Mora MC, Gomez L (1993). Field trial of vaccination against American trypanosomiasis (Chagas' disease) in dogs. Am J Trop Med Hyg.

[32] Perez Brandan C, Padilla AM, Xu D, Tarleton RL, Basombrio MA (2011). Knockout of the *dhfr-ts* gene in *Trypanosoma cruzi* generates attenuated parasites able to confer protection against a virulent challenge. PLoS Negl Trop Dis.

[33] Sanchez-Valdez FJ, Perez Brandan C, Ramirez G, Uncos AD, Zago MP, Cimino RO, Cardozo RM, Marco JD, Ferreira A, Basombrio MA (2014). A monoallelic deletion of the TcCRT gene increases the attenuation of a cultured *Trypanosoma cruzi* strain, protecting against an in vivo virulent challenge. PLoS Negl Trop Dis.

[34] Basombrío M, Besuschio S (1982). *Trypanosoma cruzi* culture used as vaccine to prevent chronic Chagas' disease in mice. Infect Immun.

[35] Cardillo F, Voltarelli JC, Reed SG, Silva JS (1996). Regulation of Trypanosoma cruzi infection in mice by gamma interferon and interleukin 10: role of NK cells. Infect Immun.

[36] Marinho CR, Nunez-Apaza LN, Martins-Santos R, Bastos KR, Bombeiro AL, Bucci DZ, Sardinha LR, Lima MR, Alvarez JM (2007). IFN-gamma, but not nitric oxide or specific IgG, is essential for the in vivo control of low-virulence Sylvio X10/4 Trypanosoma cruzi parasites. Scand J Immunol.

[37] Snapper CM, Paul WE (1987). Interferon-gamma and B cell stimulatory factor-1 reciprocally regulate Ig isotype production. Science.

[38] Umekita LF, Mota I (1989). Role of the mononuclear phagocytic system in the immune and nonspecific clearance of Trypanosoma cruzi bloodstream trypomastigotes. Braz J Med Biol Res.

[39] Brodskyn CI, Silva AM, Takehara HA, Mota I (1989). IgG subclasses responsible for immune clearance in mice infected with Trypanosoma cruzi. Immunol Cell Biol.

[40] Machado FS, Martins GA, Aliberti JC, Mestriner FL, Cunha FQ, Silva JS (2000). Trypanosoma cruzi-infected cardiomyocytes produce chemokines and cytokines that trigger potent nitric oxide-dependent trypanocidal activity. Circulation.

[41] Marinho CR, Bastos KR, Sardinha LR, Grisotto MG, Lima MR, Alvarez JM (2004). Challenge of Trypanosoma cruzi chronically infected mice with trypomastigotes activates the immune system and reduces subpatent parasitemia levels. J Parasitol.

[42] Lykens JE, Terrell CE, Zoller EE, Divanovic S, Trompette A, Karp CL, Aliberti J, Flick MJ, Jordan MB (2010). Mice with a selective impairment of IFN-gamma signaling in macrophage lineage cells demonstrate the critical role of IFN-gamma-activated macrophages for the control of protozoan parasitic infections in vivo. J Immunol.

[43] Aliberti JC, Souto JT, Marino AP, Lannes-Vieira J, Teixeira MM, Farber J, Gazzinelli RT, Silva JS (2001). Modulation of chemokine production and inflammatory responses in interferon-gamma- and tumor necrosis factor-R1-deficient mice during *Trypanosoma cruzi* infection. Am J Pathol.

[44] Dos Santos VF, Pontes C, Dominguez MR, Ersching J, Rodrigues MM, Vasconcelos JR (2014). CD8(+) T cell-mediated immunity during *Trypanosoma cruzi* infection: a path for vaccine development?. Mediat Inflamm.

