# Supplementary figures and images for: Effects of IFN-γ coding plasmid supplementation in the immune response and protection elicited by Trypanosoma cruzi attenuated parasites
Source: BMC Infect Dis. 2017 Nov 25;17:732. doi: 10.1186/s12879-017-2834-6 (PMC5702110; doi:10.1186/s12879-017-2834-6)

## INTRAMUSCULAR

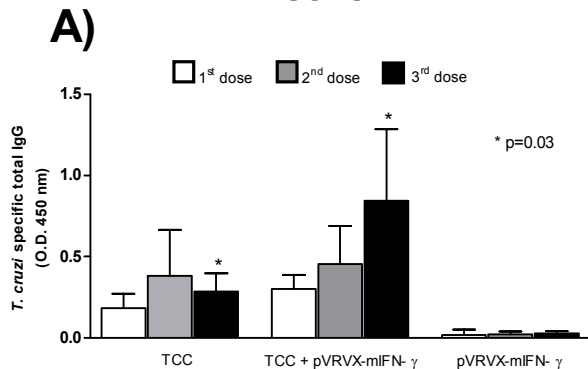

## ORAL

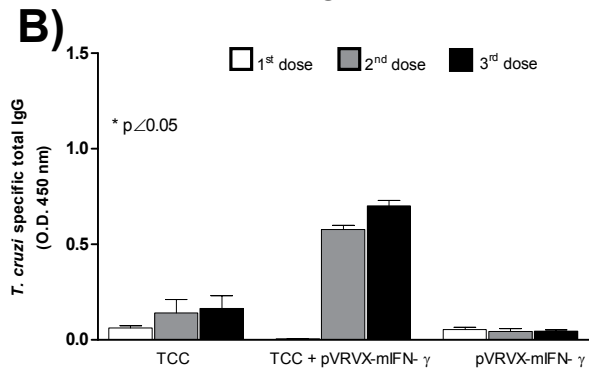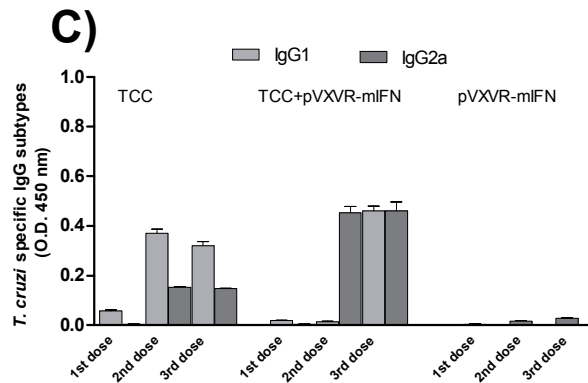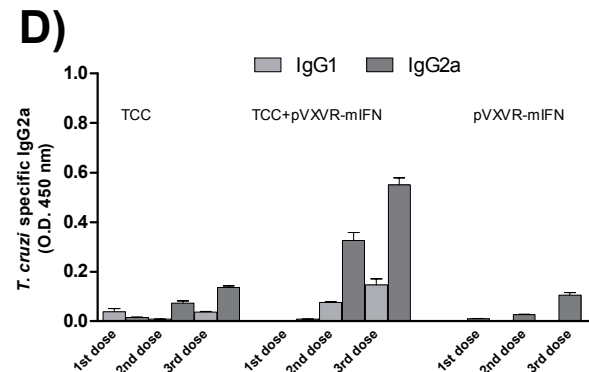

Supplement: Additional file 1: Figure S1. — pVXVR-mIFN-γ administration alters the parasite-specific immune response elicited by infection with attenuated parasites by the intramuscular and oral route. Mice (n = 4) were infected (4 weeks apart) with 3 doses of 105 metacyclic trypomatigotes of the attenuated TCC strain, 50 μg of plasmid pVXVR-mIFN-γ or a combination of both by the intramuscular (A-C) or oral (B-D) route. After each infection dose, serum samples were collected for (A-B) T. cruzi specific total IgGs levels and (C-D) serum levels of parasite specific IgG subtypes. (PDF 48 kb) [file 12879_2017_2834_MOESM1_ESM.pdf]
